# Substitution of Soy Protein for Casein Prevents Oxidative Modification and Inflammatory Response Induced in Rats Fed High Fructose Diet

**DOI:** 10.1155/2014/641096

**Published:** 2014-04-15

**Authors:** S. Sreeja, Rajagopalan Geetha, Emayavaramban Priyadarshini, Krishnamoorthy Bhavani, Carani Venkatraman Anuradha

**Affiliations:** ^1^Department of Biochemistry and Biotechnology, Annamalai University, Annamalai Nagar, Tamil Nadu 608002, India; ^2^Department of Pathology, Mahatma Gandhi Medical College and Research Institute, Puducherry 607402, India

## Abstract

Fructose-rich diet is known to cause metabolic dysregulation, oxidative stress, and inflammation. We aimed to compare the effects of two dietary proteins of animal and plant origins on fructose-induced oxidative stress and inflammatory changes in liver. Wistar rats were fed either starch or fructose (60%) diet with casein or soy protein (20%) as the protein source for 8 weeks. Glucose and insulin, glycated hemoglobin and fructosamine, AOPP, and FRAP were determined in circulation. Intracellular ROS, oxidatively modified proteins (4-HNE and 3-NT adducts), adiponectin, TNF-**α**, IL-6 and PAI-1 mRNA expression, phosphorylation and activation of JNK and IKK**β**, and NF-**κ**B binding activity were assayed in liver. In comparison with starch fed group, fructose + casein group registered significant decline in antioxidant potential and increase in plasma glucose, insulin, and glycated proteins. Increased ROS production, 4-HNE and 3-NT modified proteins, JNK and IKK**β** activation, and NF-**κ**B binding activity were observed in them along with increased gene expression of PAI-1, IL-6, and TNF-**α** and decreased adiponectin expression. Substitution of soy protein for casein reduced oxidative modification and inflammatory changes in fructose-fed rats. These data suggest that soy protein but not casein can avert the adverse effects elicited by chronic consumption of fructose.

## 1. Introduction

The consumption of fructose has increased greatly due to the widespread use of high fructose corn syrup as a sweetening agent in food industry. This has received the attention of many investigators since obesity and several aspects of the metabolic syndrome, such as hypertension, dyslipidemia, and glucose intolerance, are strongly correlated with the consumption of high fructose diet. Studies have confirmed that high fructose feeding causes alterations in glucose and lipid metabolism, a substantial decrease in peripheral insulin sensitivity, glucose intolerance, and hypertension [1, 2]. In support of this, fructose-fed rodents are widely used as a dietary model of metabolic syndrome, insulin resistance, and type 2 diabetes.

Diet can have an impact on the balance between pro- and antioxidants and also between pro- and anti-inflammatory cytokines. For example, chronic consumption of fructose has been shown to stimulate reactive oxygen species (ROS) production, lower antioxidant power and initiate proinflammatory processes, and cause dysregulation of adipokines [3]. ROS induce direct damage to cellular components by oxidizing lipids, proteins, or DNA. 4-Hydroxy-2,3-nonenal (HNE) and 4-hydroxy-2,3-alkenals of different chain length originate as a consequence of peroxidation of lipids. These molecules form aldehyde-protein adducts by interacting with reactive groups of cysteine, lysine, and histidine which produce damage to cells and tissues [4]. Nitration of tyrosine residues of proteins occurs during excessive formation of peroxynitrite [5]. 3-Nitrotyrosine (3-NT) modified protein serves as a marker of nitrooxidative stress. 4-HNE and 3-NT formation alter protein structure and function and can have cytotoxic effects [4]. Increased ROS also activate a redox-sensitive transcription factor, namely, nuclear factor kappa B (NF-*κ*B) and c-Jun N-terminal kinases (JNK), a member of mitogen activated protein kinase (MAPK) family which plays a major role in inflammatory signaling [6].

Dietary carbohydrates, rather than fat, activate proinflammatory processes through their effect on the fatty acid composition of lipids and membranes [7]. There is compelling evidence that fructose diet can increase the production of proinflammatory cytokines such as TNF-*α*, plasminogen-activator inhibitor (PAI)-1, and interleukin (IL)-6 and decrease that of adiponectin, an anti-inflammatory cytokine [8, 9].

During chronic consumption of fructose, endogenous antioxidants may be overwhelmed to prevent ROS-induced damage and therefore diet-derived or supplemented antioxidants could be important to maintain health. Dietary proteins, regarded as a source of nitrogen and essential amino acids, are needed for growth and maintenance of physiological functions. Casein, a milk phosphoprotein, supplies amino acids, carbohydrates, and the two inorganic elements, calcium and phosphorus. Bioactive peptides from milk proteins have been reported to have potential health benefits [10]. Soy protein, a plant derived protein, contains chemical constituents like isoflavones, saponins, and phytic acid of which isoflavones exhibit strong antioxidant property. Bioactive peptides and isoflavones from soy proteins have been shown to have antiatherosclerotic, antidiabetic, and hypolipidemic effects [11]. However, the role of these two proteins on inflammatory changes induced by high fructose is not very well explored. We designed this study to investigate the differential effects of dietary casein and soy protein on oxidative stress and inflammation when administered along with high fructose.

## 2. Methods

### 2.1. Fine Chemicals, Kits, and Solvents

Defatted soy protein and fat-free casein were procured from Sakthi Sugars Limited, Coimbatore, India, and Sisco Research Laboratory, Mumbai, India, respectively. Insulin and glucose assay kits were obtained from Monobind Microwells Inc, CA, USA, and Agappe Diagnostics Pvt. Ltd, Kerala, India, respectively. Antibodies such as phospho (T183/Y185) JNK and phospho-IKK*α*/*β* (Ser176/180) and anti-IKK*β* were purchased from Cell Signaling Technology, MA, USA. Anti-JNK was obtained from Santacruz Biotechnology, CA, USA. Enhanced chemiluminescence (ECL, Immobilon HRP Western Substrate) kit was purchased from Thermo Scientific, MA, USA. Nuclear extraction kit and NF-*κ*B p65 transcription factor assay kit were bought from Cayman Chemical Company, MI, USA. Supersensitive polymer-horseradish peroxidase immunohistochemistry detection kit was purchased from Biogenex laboratories, San Ramon, CA, USA. Antibodies, namely, anti-3-NT and anti-4-HNE, were purchased from Invitrogen, USA, and Merck (Calbiochem), Darmstadt, Germany, respectively. Primers were purchased from Sigma-Aldrich, MO, USA, and the SYBR Green-qPCR master mix was purchased from Thermo Scientific, MA, USA.

### 2.2. Experimental Design and Diet

Animals were maintained and cared for according to the guidelines of the Institutional Animal Ethics Committee (IAEC), Rajah Muthiah Medical College and Hospital, Annamalai Nagar, and all the experiments were approved by the IAEC (no. 160/1999/CPCSEA/770). Adult male albino Wistar rats weighing 140–160 g were individually housed under hygienic conditions in polypropylene cages under 12 hr light/12 hr dark cycle (at 22–24°C). After acclimatization for a period of 1 week, the rats were randomized into four groups containing 6 animals each. The animals received any one of the semisynthetic diets varying in carbohydrate and protein sources (Table 1)* ad libitum*. At the end of the 8 weeks, the rats were overnight fasted and sacrificed by cervical decapitation. Blood and liver tissues were collected and processed for further study.

### 2.3. Biochemical Parameters

Plasma glucose and insulin were assayed using kits. Glycated hemoglobin and fructosamine were evaluated in all blood and plasma, respectively, by the methods outlined elsewhere [12]. Plasma levels of advanced oxidation protein products (AOPP) [13] and the total antioxidant potential, ferric reducing ability of plasma (FRAP) [14], and the intracellular levels of ROS were measured [15].

### 2.4. Immunoblotting

Liver homogenate was processed and subjected to immunoblotting by the procedure outlined elsewhere [16]. The protein abundance of phospho (T183/Y185) JNK and phospho-IKK*α*/*β* (Ser176/180) and their respective total forms were analyzed by immunoblotting. The protein bands were visualized by enhanced chemiluminescence detection using Immobilon HRP Western Substrate, and finally the densitometric analysis of the bands was performed using Image J software (National Institute of Health Bethesda, MD, USA).

### 2.5. NF-*κ*B p65 Factor Binding Assay

The extraction of hepatic nuclear fraction and the detection of the levels of NF-*κ*B p65 in the nuclear fraction were done using nuclear extraction kit and transcription factor assay kit, respectively. NF-*κ*B contained in the nuclear extract was allowed to bind specifically to a specific double stranded DNA containing NF-*κ*B response element, immobilized in the wells of a 96-well plate. The binding of NF-*κ*B to DNA was visualized by means of anti-NF-*κ*B p65 antibody, which specifically recognizes activated NF-*κ*B. A secondary antibody conjugated to horseradish peroxidase was added to provide a sensitive colorimetric readout at 450 nm. The specificity of nuclear factor activation was determined by competition experiments using wild-type and mutant consensus oligonucleotides provided with the kit.

### 2.6. RNA Preparation and Real-Time Polymerase Chain Reaction (qRT-PCR) Analysis

Total cellular RNA was extracted from rat liver using TriZol reagent. RNA concentration was determined spectrophotometrically at 260 nm (Biophotometer plus, Eppendorf, Hamburg, Germany) and the purity of RNA preparation was checked by calculating the absorbance ratio at 260/280 nm. qRT-PCR was conducted in a two-step PCR procedure. Total cellular RNA (2.0 *μ*g) was reverse transcribed by standardized procedure and the transcribed cDNA was quantified (Biophotometer Plus, Eppendorf, Hamburg, Germany). qRT-PCR amplification was performed in a 20 *μ*L reaction mixture containing cDNA (100 ng), 1 *μ*L each of 0.3 *μ*M of reverse and forward primers, 10 *μ*L Maxima SYBR green qPCR master mix, and sterile water. The nucleotide sequences of primers used are given in Table 2. PCR program was conducted using real-time PCR system Mastercycler ep realplex (Eppendorf, Hamburg, Germany) in universal cycling conditions (10 min at 95°C, 40 cycles of 2 min at 95°C, 30 sec at 60°C (or the optimal Tm), and 20 sec at 72°C). The amount of the target gene normalized to an endogenous control glyceraldehyde 3 phosphate dehydrogenase (GAPDH) by 2^−ΔΔCT^ method and the relative quantity was expressed in bar graphs as fold change with respect to control.

### 2.7. Immunohistochemistry

For immunohistochemistry, 4 *μ*m paraffin liver sections were deparaffinized with xylene and rehydrated with graded concentrations of isopropyl alcohol. Separated sections were processed. Slides were incubated overnight with anti-4 HNE or 3-NT antibody (1 : 200 dilution). The slides were rinsed well with phosphate buffer and incubated with super enhancer reagent for 30 min. After rinsing with phosphate buffer, incubation was done with supersensitive polymer-horseradish peroxidase immunohistochemistry detection system. Sections were washed with buffer and incubated with a DAB solution for 5 min. Sections were counterstained with hematoxylin and observed under the light microscope.

### 2.8. Statistical Analysis

The values are given as means ± SD with *n* = 6 for biochemical analysis and *n* = 4 for immunoblot and qRT-PCR analysis. Statistical significance was done by one-way analysis of variance (ANOVA) followed by Tukey's test. The effects of varying carbohydrate and protein sources and their interaction were determined by two-way ANOVA using GraphPad Prism version 6.0 (USA). A value of *P* < 0.05 was considered significant for all cases.

## 3. Results

### 3.1. Biochemical Parameters

Significant increases in the levels of glucose, insulin, fructosamine, and glycated hemoglobin were observed in the fructose + casein group compared to starch + casein group. Soy substitution in place of casein significantly decreased the fasting glucose and insulin levels and the levels of glycated hemoglobin and fructosamine. The type of protein either casein or soy protein did not affect these parameters when given along with starch (Table 3).

Fructose + casein group showed higher ROS and AOPP levels and decreased levels of FRAP. Elevated levels of ROS and AOPP were reduced, and the concentrations of FRAP levels were within normal limits in fructose + soy group. The protein and carbohydrate sources showed independent and interactive effects on ROS, AOPP, and FRAP levels. ROS, AOPP, and FRAP levels did not differ significantly between the two protein groups fed starch diet (Table 3).

### 3.2. Biomarkers of Oxidative Stress

**Figure 1:**
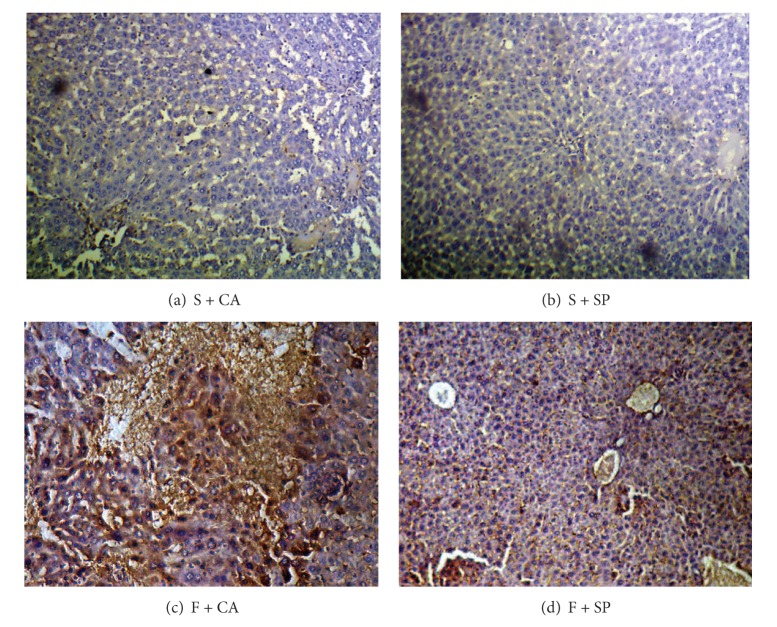
Immunohistochemical detection of 4-HNE adducts (10x). ((a) and (b)) No apparent differences in immunoreactivity were observed in S + CA and S + SP groups. (c) Cytoplasmic immunostaining was more distinct in F + CA group compared to other groups. (d) Decreased intensity of immunostaining was observed in F + SP.

**Figure 2:**
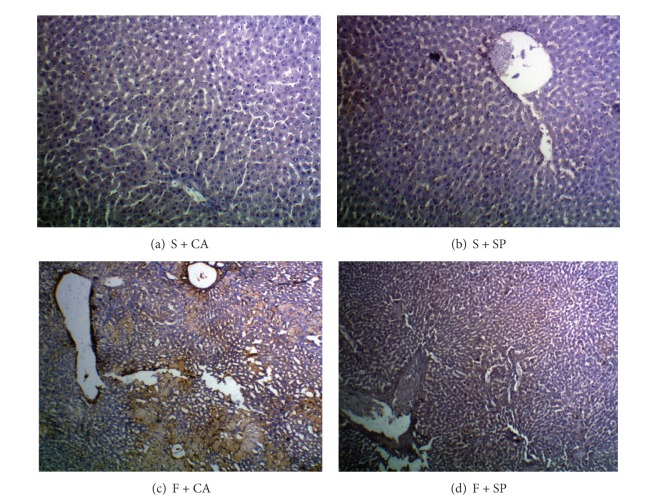
Detection of 3-NT adducts by immunohistochemistry (10x). ((a) and (b)) No immunoreactivity was observed in S + CA and S + SP groups. (c) F + CA group showed more immunopositivity for 3-NT adducts in the liver cytoplasm. (d) Marked reduction of 3-NT adducts was seen in the liver of F + SP group.

The immunohepatic localization of 4-HNE and 3-NT modified protein adducts is given in Figures 1 and 2. In fructose + casein (F + CA) group, intense cytoplasmic staining of 4-HNE and 3-NT staining were observed compared to starch + casein (S + CA) group. Substantial reduction in immunoreactivity for both 4-HNE and 3-NT was observed in the liver of fructose-fed rats given soy protein (F + SP). The starch groups fed casein or soy protein showed no difference in the immunoreactivity as compared to each other.

### 3.3. mRNA Expression of TNF-*α*, IL-6, PAI-1, and Adiponectin

**Figure 3:**
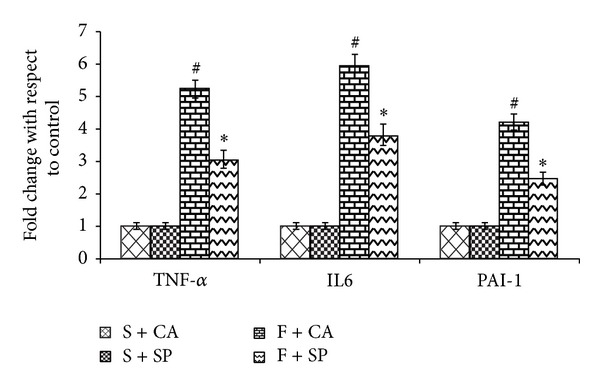
mRNA expression of inflammatory cytokines. Significant elevations of TNF-*α*, IL-6, and PAI-1 gene expression were observed in F + CA group, compared to F + SP and S + CA groups. Soy protein substitution for casein significantly reduced TNF-*α*, IL-6, and PAI-1 expression in fructose-fed rats. S + SP and S + CA groups show no apparent differences in the mRNA levels of inflammatory cytokines. Values are means ± SD (*n* = 4). Bars with different symbols denote significance (*P* < 0.05) with respect to the starch fed counterparts.

**Figure 4:**
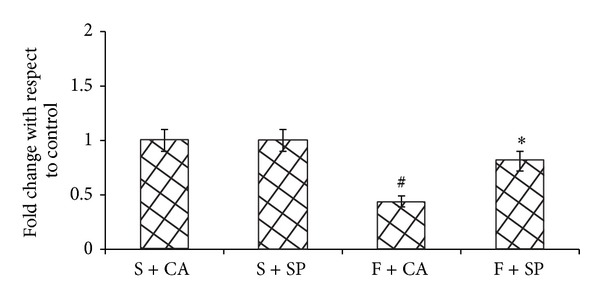
Adiponectin expression. F + SP group showed improved adiponectin expression whereas F + CA decreased the adiponectin expression when compared to the starch fed counterpart. Values are means ± SD (*n* = 4). Bars with different symbols denote significance (*P* < 0.05) with respect to the starch fed counterparts.

Figures 3 and 4 show the mRNA levels of inflammatory cytokines and adiponectin in liver. The gene expression of adiponectin was decreased and that of TNF-*α*, IL-6, and PAI-1 expression was increased in the liver of fructose and casein fed group (F + CA) as compared to its starch + casein fed group (S + CA). In fructose-fed group, soy protein addition in place of casein improved the expression of adiponectin and prevented the increase in the expression of TNF-*α*, IL-6, and PAI-1 (F + SP). No significant changes in the mRNA expression of inflammatory cytokines and adiponectin were observed in the starch fed groups irrespective of protein variation.

### 3.4. Activation of Inflammatory Signals

**Figure 5:**
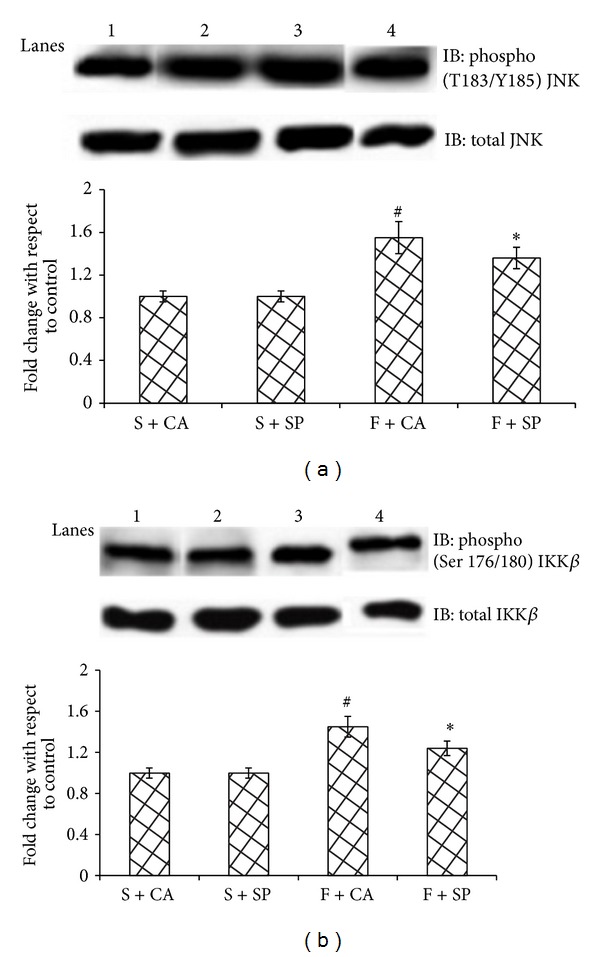
Immunoblots and representative graphs of JNK and IKK*β*. Lane 1: S + CA, Lane 2: S + SP, Lane 3: F + CA, and Lane 4: F + SP. F + CA group shows increased phosphorylation of (a) JNK and (b) IKK*β* compared to the starch fed counterparts. Soy protein substitution for casein (F + SP group) reduced the protein abundance of inflammatory kinases. Values are means ± SD (*n* = 4). Bars with different symbols denote significance (*P* < 0.05) with respect to the starch fed counterparts.

**Figure 6:**
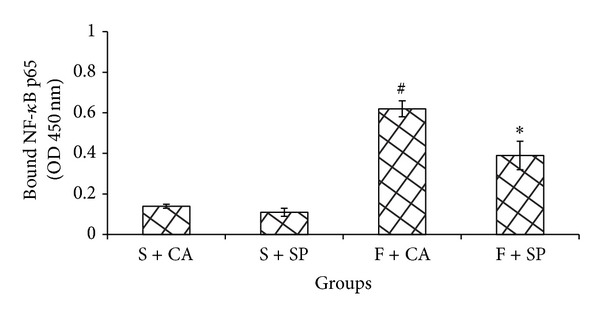
Levels of  NF-*κ*B in the nuclear fraction. Increased binding activity of NF-*κ*B was seen in F + CA group when compared to all other groups. NF-*κ*B levels in the nuclear fraction were significantly reduced in F + SP group when compared to F + CA group. Values are means ± SD (*n* = 4). Bars with different symbols denote significance (*P* < 0.05) with respect to the starch fed counterparts.

When compared with starch + casein group, fructose + casein fed rats significantly increased the phosphorylation status of hepatic JNK and IKK*β* (Figures 5(a) and 5(b)) and the levels of nuclear NF-*κ*B p65 (Figure 6). The increase was brought down significantly in fructose + soy protein fed group. Starch fed groups showed no significant differences amongst each other.

## 4. Discussion

The findings reported here demonstrate that fructose + casein diet induced ROS generation, increased the accumulation of oxidatively modified proteins and the expression of proinflammatory cytokines, and activated NF-*κ*B signaling, while soy protein substitution reduced the damaging effects of fructose and protected the cells.

ROS generation due to fructose consumption can be attributed to unregulated glycolytic pathway, depletion of ATP due to excess fructose metabolism, increased lipid levels, and an increased flux through the Krebs cycle [17]. Consistent with previous reports [18, 19], fructose feeding elevated intracellular ROS and AOPP levels. AOPP indicates the extent of oxidative damage to circulating proteins especially albumin and is measured by reaction between plasma protein and chlorinated oxidants [20]. The total antioxidant power of plasma is assayed as a function of ferric reducing ability of plasma and is contributed by the nonenzymic antioxidants of plasma [14]. Decline in the FRAP value is responsible for the elevated intracellular levels of ROS and plasma AOPP levels in fructose + casein group. The presence of hepatic oxidative stress correlates with immunopositivity for oxidatively modified proteins (4-HNE and 3-NT adducts) which are considered as biomarkers of oxidative damage.

Activation of NF-*κ*B through IKK*β* and stimulation of JNK have been observed in fructose-fed rats. JNK activation is believed to play an important role in serine phosphorylation of insulin receptor substrate-1 and to decrease insulin signaling. Rat primary hepatocytes exposed to fructose show increased JNK activity and decreased PI3K/Akt pathway [21]. Isoforms of JNK are activated in human hepatic stellate cells exposed to HNE [22]. ROS can activate JNK1 by oxidative modification and inactivation of MAPK phosphatases which are negative regulators of JNK. IKK*β* activation is responsible for activation of the NF-*κ*B/Rel family of transcription factors. IKK*β* phosphorylation and higher levels of NF-*κ*B p65 in the nuclear fraction of fructose + casein fed group indicate nuclear translocation of NF-*κ*B p65. The production of proinflammatory cytokines such as TNF-*α* and IL6 by lipid loaded liver cells can be activated by NF-*κ*B which in turn promotes JNK signaling. The proinflammatory kinases JNK1 and IKK*β* are activated by almost all the signaling pathways proposed to cause insulin resistance and interference with either JNK1 or IKK*β* activity has been found to be protective in lipotoxic conditions and improve insulin signaling in mouse models of obesity and lipid-induced glucose intolerance [23, 24].

It has been postulated that dietary factors can regulate the expression of genes directly, by regulating the activity of certain transcription factors that control the expression of genes involved in specific metabolic pathways. Fructose feeding also stimulated the gene expression of proinflammatory cytokines, namely, TNF-*α*, IL6, and PAI-1, and decreased the expression of adiponectin. Decreased adiponectin levels and bioactivity have been reported in obesity and obesity-related complications, including insulin resistance, diabetes, cardiovascular diseases, and nonalcoholic fatty liver disease (NAFLD). Furthermore, hyperinsulinemia significantly lowers plasma adiponectin levels under euglycemic conditions [25]. TNF-*α* and IL-6 are the cytokines that are known to be expressed during inflammatory condition and are elevated in obesity-related inflammatory diseases. PAI-1 mRNA expression in the liver has been shown to be higher in NAFLD patients and high levels of PAI-1 can result from released PAI-1 from endothelial cells due to several stimuli like high glucose, cytokines (TNF-*α*), and growth factors. Increase in PAI-1 contributes to lipid accumulation, macro- and microvascular complications, and hepatic inflammation [26].

The type of protein consumed has an impact on oxidative stress and inflammatory signaling. It is observed in this study that soy protein when included in fructose diet instead of casein reduced ROS generation and prevented the activation of JNK and NF-*κ*B and the expression of TNF-*α*, IL-6, and PAI-1 but at the same time increased the adiponectin expression. The amino acid composition of soy protein may play an important role in its biological effects [27]. The presence of higher levels of arginine, glycine, and cysteine in soy protein than casein may have a beneficial role in reducing oxidative stress. Studies show that dietary glycine exerts protective effects by preventing oxidative stress and by blocking posttranslational inhibition of antioxidant enzymes such as manganese-superoxide dismutase, TNF-*α* production, and iNOS overexpression against liver injury and death caused by hemorrhagic shock [28]. Improvement in the antioxidant glutathione status after exercise is related to cysteine content of dietary proteins [29]. Arginine has been found to have antioxidative and antiatherogenic functions and is rich in soy protein when compared to casein [30].

Besides the amino acid composition, isoflavones such as genistein, daidzein, glycitein, and their glycosides, glycoside malonates, and glycoside acetates of soy protein could have contributed to its beneficial effects. The reduction of oxidative stress by isoflavones from soy has been shown in several studies. For example, genistein protects endothelial cells from oxidative stress induced by hydrogen peroxide [31]. Diet rich in genistein and daidzein has been shown to inhibit free radical formation, lipid peroxidation, and oxidative stress in human lymphocytes isolated from healthy subjects by inhibiting TNF-*α* induced NF-*κ*B activation [32].

The differential effects of soy and casein were seen only in fructose-fed rats but not when starch was used as the dietary carbohydrate. It has been suggested that the quality of dietary proteins when combined with carbohydrate intake may affect muscle protein kinetics by stimulating protein synthesis and by reducing protein breakdown. Further, differences between the effects of soy and casein protein may be more pronounced under conditions like elevated protein turnover, resistance exercise, and hyperinsulinemia [33].

## 5. Conclusion

In conclusion, casein when given along with fructose failed to attenuate oxidative stress and the activation of inflammatory signals, whereas soy protein replacement reduced oxidative damage and had profound anti-inflammatory response against the detrimental effects of fructose. A comparison of the efficacy of liver in the two protein groups fed fructose needs to be analyzed through liver function tests. However, from the present findings it can be suggested that soy protein may be included as one of the dietary agents that can prevent or overturn the hepatic inflammation and oxidative stress associated with metabolic syndrome resulting from consumption of sugar-enriched diet.

## Figures and Tables

**Table 1:** Diet composition.

	Starch diet		Fructose diet	
	Casein protein (S + CA)	Soy protein (S + SP)	Casein protein (F + CA)	Soy protein (F + SP)
Corn starch	60.0	60.0	—	—
Fructose	—	—	60.0	60.0
Casein (fat free)	20.0	—	20.0	—
Soy protein	—	20.0	—	20.0
Groundnut oil	5.0	5.0	5.0	5.0
Wheat bran	11.0	11.0	11.0	11.0
Salt mixture*	3.5	3.5	3.5	3.5
Vitamin mixture^*ψ*^	0.5	0.5	0.5	0.5

*The composition of mineral mix (g/kg): MgSO_4_. 7H_2_O-30.5; NaCl 65.2; KCl-105.7; KH_2_PO_4_-200.2; MgCO_3_-3.65; Mg(OH)_2_·3H_2_O-38.8; FeC_6_H_5_O_7_·5H_2_O-40.0; CaCO_3_-512.4; KI-0.8; NaF-0.9; CuSO_4_·5H_2_O-1.4; MnSO_4_-0.4; and CONH_3_-0.05.

^Ψ^The composition of vitamin mix (g/kg): thiamine mono nitrate-3; riboflavin-3; pyridoxine HCl-3.5; nicotinamide-15; D-calcium pantothenate-8; folic acid-1; D-biotin-0.1; cyanocobalamin-0.005; vitamin A acetate-0.6; *α*-tocopherol acetate-25; and choline chloride-10.

**Table 2:** List of genes and primer sequences.

Gene	ID	NCBI reference sequence	Official symbol	Forward primer sequence (5′-3′)	Reverse primer sequence (5′-3′)
Glyceraldehyde-3-phosphate dehydrogenase	24383	NC_005103.3	GAPDH	aaggggaacccttgatatgg	cggagatgatgacccttttg
Tumor necrosis factor	24835	NC_005119.3	TNF-*α*	gctgaggttggacggataaa	aaaatcctgccctgtcacac
Interleukin 6	24499	NC_005103.3	IL6	caaaagagagcctgggactg	ggctgaagaattgctggaag
Plasminogen-activator inhibitor type 1	24617	NC_005111.3	PAI-I	gatctcctgggatcactcca	ttgggggatgtctacatggt
Adiponectin	246253	NC_005110.3	Adipoq	gacaaggccgttctcttcac	gtccccttccccatacactt

**Table 3:** Biochemical parameters.

Parameters	S + CA	S + SP	F + CA	F + SP	Two-way ANOVA		
					CARB	PROT	INTER
Glucose (mg/dL)	92.4 ± 5.8^a^	86.4 ± 4.1^a^	202.5 ± 16.9^b^	112.8 ± 9.51^c^	*P* < 0.0001	*P* < 0.0001	*P* < 0.0001
Insulin (*μ*IU/mL)	17.2 ± 0.7^a^	16.9 ± 0.8^a^	30.5 ± 2.4^b^	19.3 ± 1.5^c^	*P* < 0.0001	*P* < 0.0001	*P* < 0.0001
Glycated hemoglobin (mg/g Hb)	0.35 ± 0.01^a^	0.34 ± 0.02^a^	0.83 ± 0.04^b^	0.40 ± 0.02^d^	*P* < 0.0001	*P* < 0.0001	*P* < 0.0001
Fructosamine (mmol/L)	0.47 ± 0.02^a^	0.48 ± 0.03^a^	1.3 ± 0.10^b^	0.54 ± 0.03^a^	*P* < 0.0001	*P* < 0.0001	*P* < 0.0001
AOPP (*μ*mol/L)	90.6 ± 4.6^a^	65.8 ± 2.8^a^	160.5 ± 8.2^b^	110.18 ± 5.3^c^	*P* < 0.0001	*P* < 0.0001	*P* < 0.0001
FRAP (*μ*mol/L)	990.3 ± 3.8^a^	1076.2 ± 2.5^a^	770.4 ± 3.3^b^	956.6 ± 3.9^c^	*P* < 0.0001	*P* < 0.0001	*P* < 0.0001
Intracellular ROS generation in liver (mean fluorescence intensity)	343 ± 2.7^a^	339 ± 1.8^a^	583 ± 4.1^b^	352 ± 2.3^c^	*P* < 0.0001	*P* < 0.0001	*P* < 0.0001

Data are means ± SD of 6 rats from each group. S + CA: starch + casein diet-fed rats; S + SP: starch + soy protein diet-fed rats; F + CA: fructose + casein diet-fed rats; F + SP: fructose + soy protein diet-fed rats. NS: not significant; CARB: carbohydrate; PROT: protein; INTER: interaction; ROS: reactive oxygen species; AOPP: advanced oxidation protein products; FRAP: ferric reducing antioxidant power. Values that bear different superscripts are significantly different from each other [one-way ANOVA followed by Tukey's test (*P* < 0.05)].
